# Estrogenic Activity of Mineral Oil Aromatic Hydrocarbons Used in Printing Inks

**DOI:** 10.1371/journal.pone.0147239

**Published:** 2016-01-15

**Authors:** Patrick Tarnow, Christoph Hutzler, Stefan Grabiger, Karsten Schön, Tewes Tralau, Andreas Luch

**Affiliations:** German Federal Institute for Risk Assessment (BfR), Department of Chemical and Product Safety, Berlin, Germany, Max-Dohrn-Strasse 8–10, 10598, Berlin, Germany; Seoul National University, REPUBLIC OF KOREA

## Abstract

The majority of printing inks are based on mineral oils (MOs) which contain complex mixtures of saturated and aromatic hydrocarbons. Consumer exposure to these oils occurs either through direct skin contacts or, more frequently, as a result of MO migration into the contents of food packaging that was made from recycled newspaper. Despite this ubiquitous and frequent exposure little is known about the potential toxicological effects, particularly with regard to the aromatic MO fractions. From a toxicological point of view the huge amount of alkylated and unsubstituted compounds therein is reason for concern as they can harbor genotoxicants as well as potential endocrine disruptors. The aim of this study was to assess both the genotoxic and estrogenic potential of MOs used in printing inks. Mineral oils with various aromatic hydrocarbon contents were tested using a battery of *in vitro* assays selected to address various endpoints such as estrogen-dependent cell proliferation, activation of estrogen receptor α or transcriptional induction of estrogenic target genes. In addition, the comet assay has been applied to test for genotoxicity. Out of 15 MOs tested, 10 were found to potentially act as xenoestrogens. For most of the oils the effects were clearly triggered by constituents of the aromatic hydrocarbon fraction. From 5 oils tested in the comet assay, 2 showed slight genotoxicity. Altogether it appears that MOs used in printing inks are potential endocrine disruptors and should thus be assessed carefully to what extent they might contribute to the total estrogenic burden in humans.

## Introduction

While most readers will read this article as PDF or a print-out thereof, paper- and cardboard-based printing is far from giving its swan song. The inks used can be categorized into water-based, solvent-based and mineral oil (MO)-based. With more than 420,000 t used in 2012 the latter still constitute the majority of inks used for printing in Europe and probably world-wide [1]. Applications comprise, amongst others, newspaper printing as well as the labeling and decoration of food packaging. Hence it comes as little surprise that residues of MOs are detectable in cardboard packages for food. In addition, the cardboard used is often sourced from recycled material which contains large quantities of newspaper and consequently some of its printing inks. Without further barriers the respective MOs can migrate into the packaged foodstuffs [2] and compounds from mineral oils have indeed been detected in dry foods such as rice and noodles in concentrations as high as tens to hundreds of mg/kg [3–5]. From a chemical point of view MOs consist of a complex mixture of several hundred substances. Depending on their structural features these are commonly attributed to two fractions, that is mineral oil saturated hydrocarbons (MOSHs) or mineral oil aromatic hydrocarbons (MOAHs). The former encompass naphthenes, n-alkanes and iso-alkanes, whereas the latter contain highly alkylated mono- and polycyclic aromatic hydrocarbons (PAHs) [6, 7]. The potential health hazard, particularly of MOAHs in foodstuffs is a matter of ongoing debate, which is complicated further by the fact that there are hardly any data on the individual compounds or their toxicological properties. So far the discussion has mainly focused on the potential carcinogenic properties of PAHs, the obvious reason being their tendency for DNA adduct formation subsequent to metabolic activation [8]. However, given the structural plethora of the chemical space comprised by MOAHs another concern is endocrine disruption. Yet, this point has so far not been addressed.

“Endocrine disruptors” are defined as exogenous substances or mixtures that alter functions of the endocrine system and consequently cause adverse health effects in an intact organism, or its progeny [9]. The *in vitro* assessment of such substances remains a challenge due to limited experimental accessibility of the various hormone systems by means of high throughput testing, hormone homeostasis and the multitude of signaling pathways involved. Although there has been some progress, targeted testing is still pretty much limited to the estrogenic, androgenic, thyroid and steroidogenic systems, with the majority of *in vitro* tests addressing the androgenic and estrogenic signaling cascades. Those systems rely on receptor mediated signaling cascades with xenoestrogens usually acting as ligands for the estrogen receptors (ER, subtypes α and β). Following their stimulation (*i*.*e*., by 17β-estradiol (E2) or a structural homologue) these receptors homodimerize and act as ligand activated transcription factors that bind to so called estrogen responsive elements (EREs) within the promotors of their target genes [10]. The respective signaling cascades will influence physiological processes as diverse as reproduction, bone integrity or behavior, and accordingly ERα is expressed not only in tissues with reproductive function (*e*.*g*., uterus, ovary, mammary gland, testes and prostate) but in a variety of other organs such as bone, liver, heart and brain [11]. Perturbation of ERα signaling is involved in several types of cancer [12] and while binding of xeno- or phytoestrogens does not necessarily constitute a health concern it is a potential hazard indicator [13]. The more so, as mixtures of estrogenic chemicals can have additive effects [14]. Given the high exposure of consumers against MOs used in printing inks this study therefore assessed the xenoestrogenic potential using a battery of *in vitro* assays.

## Materials and Methods

### Chemicals

Cell culture media were purchased from PAN Biotech (Aidenbach, Germany), charcoal treated fetal calf serum (FCS) was obtained from PAA (Cölbe, Germany). Substrates for the luciferase assays (D-Luciferin, ATP) and reducing agent DTT were obtained from PJK (Kleinblittersdorf, Germany). Bulk chemicals, 17β-estradiol (E2), 3-(4,5-dimethylthiazol-2-yl)-2,5-diphenyltetrazolium bromide (MTT), dichloromethane (>99.8%) and n-hexane (>97.0%) were purchased from Sigma Aldrich (Munich, Germany). Standards for MOSH analysis were n-undecane (n-C11), n-tridecane (n-C13), cyclohexyl-cyclohexane and 5α-cholestane (Cho); those for MOAH analysis were pentyl-benzene (5B), 1- and 2-methylnaphthalene (1-/2-MN), 1,3,5-tri-*tert*-butylbenzene (TBB) and perylene (Per; all from Sigma Aldrich). MOs were kind gifts from the Chemical and Veterinary Investigations Office in Stuttgart, Germany, and the Official Food Control Authority of the Canton of Zürich, Switzerland, or in house samples, respectively. Test substances for the various assays were used as provided with the exception of E2 which was dissolved in dimethyl sulfoxide (DMSO) prior to use.

### E-screen

The human mammary carcinoma MCF-7 cell line was derived from the ATCC (HTB-22). For the assay 5000 cells per well were seeded into 24-well plates using Dulbecco’s modified Eagle medium (DMEM) supplemented with 10% FCS. The cells were then allowed to attach over 6 h before being subsequently washed with hormone-free DMEM (HF-DMEM, phenol red-free DMEM supplemented with 5% charcoal stripped FCS) and subjected to hormone deprivation for another 72 h. Finally the cells were exposed to the respective MO or analytical fractions thereof for 5 days before being analyzed using cell viability as the final endpoint. Viability was assessed based on a standard MTT assay (0.5 mg/ml MTT, 2 h of incubation), using a Synergy HT plate reader from BioTek (Bad Friedrichshall, Germany) to assess the reduction of MTT to formazan at 595 nm. Data were normalized to the solvent control (set as 0%) and to 1 nM E2 treated cells (set as 100%).

### Gene expression analysis of selected genes using quantitative RT-PCR

The expression of selected genes in MCF-7 cells was analyzed by quantitative RT-PCR. Cells were seeded into 12-well plates using hormone-free medium and a concentration of 2 × 10^5^ cells per ml and well and left to settle for 48 h before being stimulated with dispersed MO or preparative fractions of MO for a total of 24 h. Following chemical treatment cells were washed in PBS and the total RNA was extracted using Trizol (Invitrogen, Carlsbad, CA, USA). The extracted RNA (1 μg) was then reversely transcribed into cDNA, using a cDNA synthesis kit (Applied Biosystems, Foster City, CA, USA) and relative transcript levels were determined in triplicate using presynthesized Taqman probes (Applied Biosystems, Foster City, CA, USA) and quantitative RT-PCR. Probes used were RPLP0 (Hs99999902_m1), CYP1A1 (Hs00153120_m1), CYP1B1 (Hs00164383_m1), PGR (Hs01556707_m1), TFF1 (Hs00907239_m1) and GREB1 (Hs00536409_m1).

### Reporter gene assay

Transactivation assays were performed as described previously [15], with some minor modifications. In brief, hERα-HeLa-9903 cells (JCRB-No. 1318, as available from the Japanese Collection of Research Bioresources Bank) were grown in a white 96-well plate in MEM supplemented with 10% charcoal treated FCS and kanamycin (60 mg/l), seeding 10^4^ cells per 100 μl and well. Cells were then left to rest for some 24 h before being stimulated with MO or E2 for another 20 h. Cellular lysis was then initiated *in situ* by adding 50 μl of lysis buffer (0.1 M tris-acetate, 2 mM EDTA, and 1% Triton-X, pH 7.8) to each well and allowed to commence for 20 minutes at room temperature and moderate shaking. The activity of any luciferase expressed was then quantified based on luminescence levels following the addition of buffer (150 μl; 25 mM glycylglycine, 15 mM MgCl_2_ and 4 mM EGTA, 1 mM DTT, 1 mM ATP, pH 7.8) and reagent solution (50 μl; 25 mM glycylglycine, 15 mM MgCl_2_ and 4 mM EGTA, 0.2 mM luciferin, pH 7.8), respectively. All values were corrected for the mean of the negative control and then related to the positive control which was set to 100%. All experiments were conducted at least in triplicate, using a Synergy HT plate reader (BioTek, Bad Friedrichshall, Germany) equipped with an automatic injector unit.

### Single-cell electrophoresis assay (comet assay)

Normal human epithelial keratinocytes (NHEKs) were isolated by overnight trypsin digestion from juvenile foreskins and subsequently cultivated in KBM-2 supplemented with bovine pituitary extract (4 μl/ml), EGF (0.125 ng/ml), insulin (5 μg/ml), hydrocortisone (0.33 μg/ml) epinephrine (0.39 μg/ml) transferrin (10 μg/ml) and CaCl_2_ (60 μM) (PromoCell, Heidelberg, Germany). For experiments, cells were then seeded in 12-well dishes and left to grow until reaching confluency. Prior to MO application the medium was changed for at least 24 h to KBM supplemented with bovine pituitary extract and CaCl_2_ only_,_ since EGF and other growth factors are known to partially repress the aryl hydrocarbon receptor (AHR) mediated response, thus interfering with the assay [16]. Exposure to test mixtures lasted for 24 h, with aphidicolin (APC, 5 μg/ml) being added after some 20 h, followed by a comet assay as described previously [17]. In brief, cells from each well were trypsinized, pelleted, redissolved in 20 μl of PBS, mixed with 300 μl of 0.5% (w/v) low melting point agarose (Cambrex,East Rutherford, NJ, USA) and applied to two coated microscope slides. The cells were then lyzed overnight at 4°C in lysis buffer (2.2 M NaCl, 8.9 mM Na_2_EDTA, 8.9 mM Tris, 10% DMSO, 0.1% Triton X-100, pH 10) followed by 20 minutes of equilibration in alkaline electrophoresis buffer (1 M NaOH, 20 mM Na_2_EDTA, pH>13) and electrophoresis for 30 minutes at 30 V and 450 mA. After neutralization in 0.4 mM Tris buffer (pH 7.5) the slides were finally dehydrated in ethanol and dried for later analysis. For comet scoring, DNA was stained with SYBR gold and the relative intensity of any DNA tails was assessed using a commercial software package (CometImager V2.2.1, MetaSystems, Altlußheim, Germany).

### Characterization of MOs by online-LC-GC-FID and GCxGC-ToF-MS

MOs were analyzed according to Biederman *et al*. using an LC-GC 9000 system (Brechbühler, Schlieren, Switzerland) equipped with a LiChrospher Si 60 LC column (250 x 20 mm i.d., 5 μm; Bischoff Analytics, Germany), a ZB-1HT Inferno GC column (15 m x 0.25 mm i.d., 0.1 μm; Phenomenex, Aschaffenburg, Germany) and an uncoated silica precolumn (10 m x 0.53 mm i.d.) [2, 3]. Separated fractions of MOAHs and MOSHs were quantified by integration using cyclohexyl-cyclohexane and 1-/2-MN as internal quantification standards for MOSHs and MOAHs, respectively.

MOAH fractions were characterized further by two dimensional GCxGC-ToF-MS using a Pegasus 4D-system (LECO, Mönchengladbach, Germany) and ultra-pure helium (purity ≥99.999%, Linde, Pullach, Germany) as carrier gas. Sample injections were performed in splitless mode using a split/splitless injector (Agilent, Waldbronn, Germany) at a constant temperature of 275°C and then subjected to separation at a constant flow of 1 ml/min using a nonpolar column (RXI-5Sil MS, 30 m x 0.25 mm i.d., 0.25 μm, Restek, Bad Homburg, Germany), and a polar column (RXI-17Sil MS, 1 m x 0.18 mm i.d., 0.18 μm, Restek) for the first and second dimension, respectively. For the first oven heating started at 70°C for 1 minutes, followed by successive ramping to 120°C (15°C/min), 170°C (8°C/min), 270°C (2°C/min) and 330°C (15°C/min, ramp held for 8 min). The same heating ramps were used for the programs of the secondary oven and the modulator, except for the use of offsets of 5°C and 15°C for oven and modulator, respectively. The program for the modulation time was as follows: 3.5 sec from the start up to 13.22 min, 4 sec from 12.22 min up to 26.62 min, 4.5 sec from 26.62 min up to 49.87 min, 8 sec from 49.89 min to 52.20 min and 10 sec from 52.20 min up to the end of the analysis. Subsequent mass analysis was performed using a temperature of 205°C for the ion source and electron impact ionisation at 70 eV. The mass range was set to 35 to 500 amu in the full scan mode with an acquisition rate of 200 full scans per second.

### Preparation of MOSH and MOAH fractions from MOs

In total 10 μl of MO were subsequently separated into MOSHs and MOAHs using the LC of the LC-GC 9000 system equipped with a LiChrospher Si 60 column, collecting the samples manually into glass vials. Fractions were eluted using a hexane (MOSHs) and dichloromethane/hexane gradient (MOAHs), respectively. For each preparation 10 μl MO were mixed with 990 μl of n-hexane, followed by 10 subsequent injections of 50 μl each using the decoupled LC-GC system for collection of the MOAH fractions into ice cold glass vials. The combined fractions were then concentrated in a rotary evaporator using 500 μl of DMSO as keeper and kept for further use in the toxicological assays.

## Results

### Estrogenic activity of MOs

The xenoestrogenic potential of MOs was initially assessed using different *in vitro* assays, namely the so called E-screen, an estrogen responsive luciferase assay and by transcriptional analysis of selected estrogen responsive genes. The first uses the estrogen-dependent proliferation of mammary carcinoma MCF-7 cells and thus provides a physiological readout, while the second and the third assay indicate interaction with ERα and activation of ER-triggered gene responses, respectively. Gene targets selected for the transcriptional assay comprised the progesterone receptor (*PGR*), trefoil factor 1 (*TFF1* or *pS2*) and the estrogen-dependent growth regulator in breast cancer 1 (*GREB1*). All genes are well established and thus frequently used estrogen response genes. The *TFF1* promoter contains a consensus ERE and two ERE half sites [18]. *GREB1* contains 3 consensus EREs in the distant promoter located 1.6–21 kb upstream the transcription start site [19]. The PGR gene contains several imperfect EREs ranging from 311 kb upstream to 4 kB downstream the transcription start site which confer to estrogen responsiveness of PGR expression [20].

**Fig 1:**
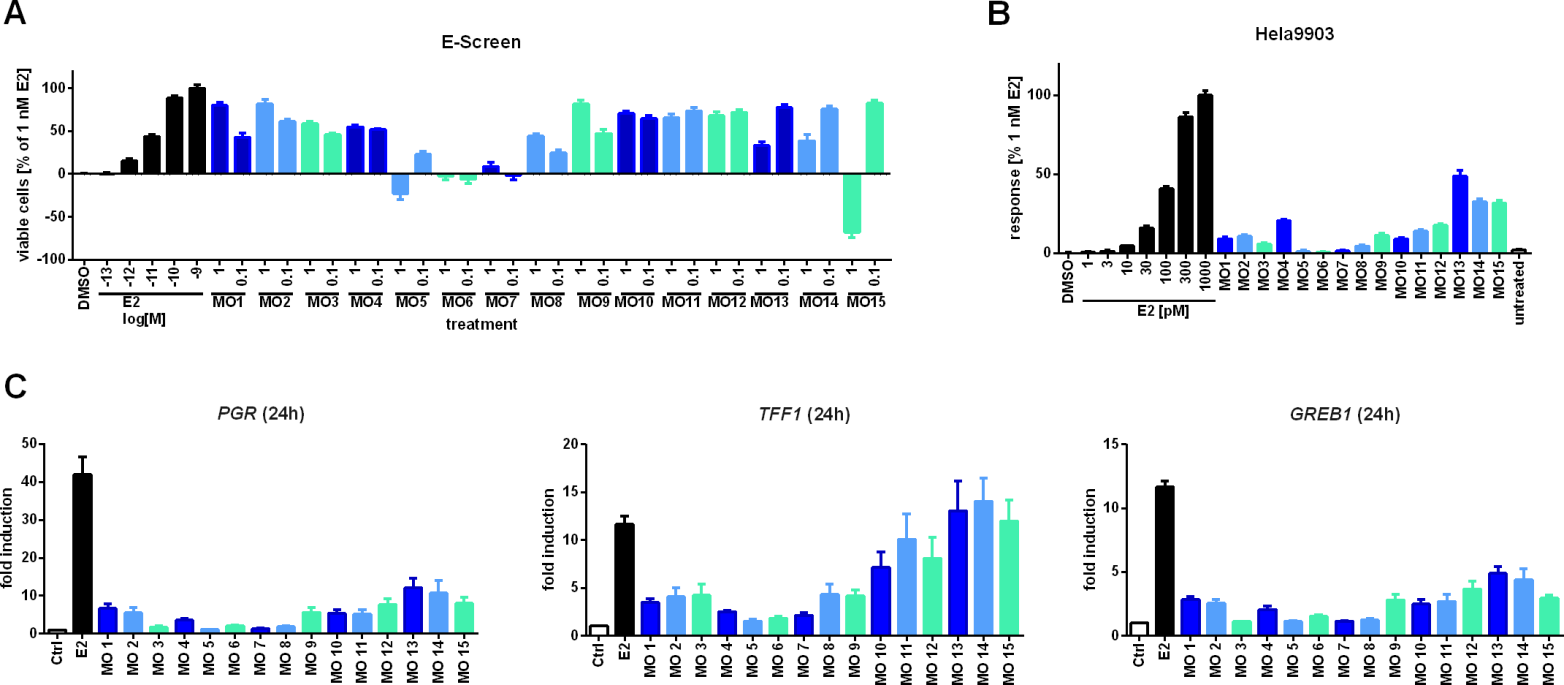
Estrogenic effects of MOs in the E-screen, the hERα-HeLa-9903 assay and induction of ER responsive transcripts as indicated. Proliferation assays with MCF-7 cells were performed subsequent to cellular stimulation with dispersions of 1 and 0.1 μl/ml MO (eq. to dil. of 1:1,000 and 1:10,000) or E2 as indicated (**A**). For the reporter gene assay hERα-HeLa-9903 cells were stimulated for 20 h with the indicated amounts of E2 or 1 μl/ml MO dispersed in medium, followed by cellular lysis and measurement of firefly luciferase activity (**B**). Transcriptional assays were following a 24-h exposure to 1 μl/ml MO or 10 nM E2, respectively (**C**). Data in all assays represent the mean ± SEM from at least three independent experiments. For the E-screen and the reporter gene assay data were corrected to accommodate the background of untreated cells and subjected to normalization using the effect of 1 nM E2. Likewise gene expression was normalized using *RPLP0* and the solvent control as references. Abbreviations: *PGR*, progesterone receptor (gene); *TFF1*, trefoil factor (gene); *GREB1*, estrogen-dependent growth regulator in breast cancer 1 (gene).

A total of 15 different MOs were tested in the E-screen at concentrations of 1 and 0.1 μl/ml (Fig 1A). All oils, except two (*i*.*e*., MO 6 and MO 7), had a notable stimulating effect on cell proliferation. For MOs 1, 2, 3, 8 and 9 stimulation of cell proliferation followed an apparent dose dependency (p<0.001 using a student’s t-test), whereas other oils such as MOs 4, 10, 11 and 12 exerted a maximal proliferative effect at both concentrations tested. Five oils were found to be cytotoxic. This included MO 6 at both concentrations, as well as MOs 5, 13, 14 and 15 at 1 μl/ml.

The xenoestrogenic potential of MOs was further investigated using hERα-HeLa-9903 cells, a well-established reporter cell line that expresses firefly luciferase under the control of a five-fold ERE (Fig 1B). The results showed a statistically significant induction of luciferase activity for all but three MOs, thus largely confirming the data from the E-screen. No luminescence induction was seen for the previously cytotoxic MOs 5 and 6 as well as the weakly proliferative MO 7. Notably, the maximum levels of induction (*i*.*e*., MO 13, 14 and 15) came close the half-maximal stimulation of the positive control, equivalent to 100 pM of E2.

Likewise the transcriptional assay confirmed estrogen-dependent gene responsiveness for several of the MOs, particularly sample numbers 1 and 2 and 9 to 15 (Fig 1C). Nevertheless, the effects varied depending on the target transcript. The mRNA levels of *PGR* and *GREB* generally featured a moderate to weak induction, while upregulation of *TFF1* was more pronounced when compared to the E2 treated positive control. Treatment with MOs 11 to 15 resulted in an induction of *TFF1* equivalent to stimulation with 10 nM E2. The results were confirmed further using two additional estrogen-dependent genes, that is *HSPB8* and *CTSD* (S2 Fig). Transcription of *HSPB8* is regulated by nongenomic estrogen responses and CTSD contains EREs located 9 kb upstream of its transcription start site [21, 22]. As with the other genes transcription was found to be affected by several of the MOs tested, inducing *HSPB8* up to twofold, and *CTSD* up to threefold (S2 Fig).

### Composition of MOs

**Fig 2:**
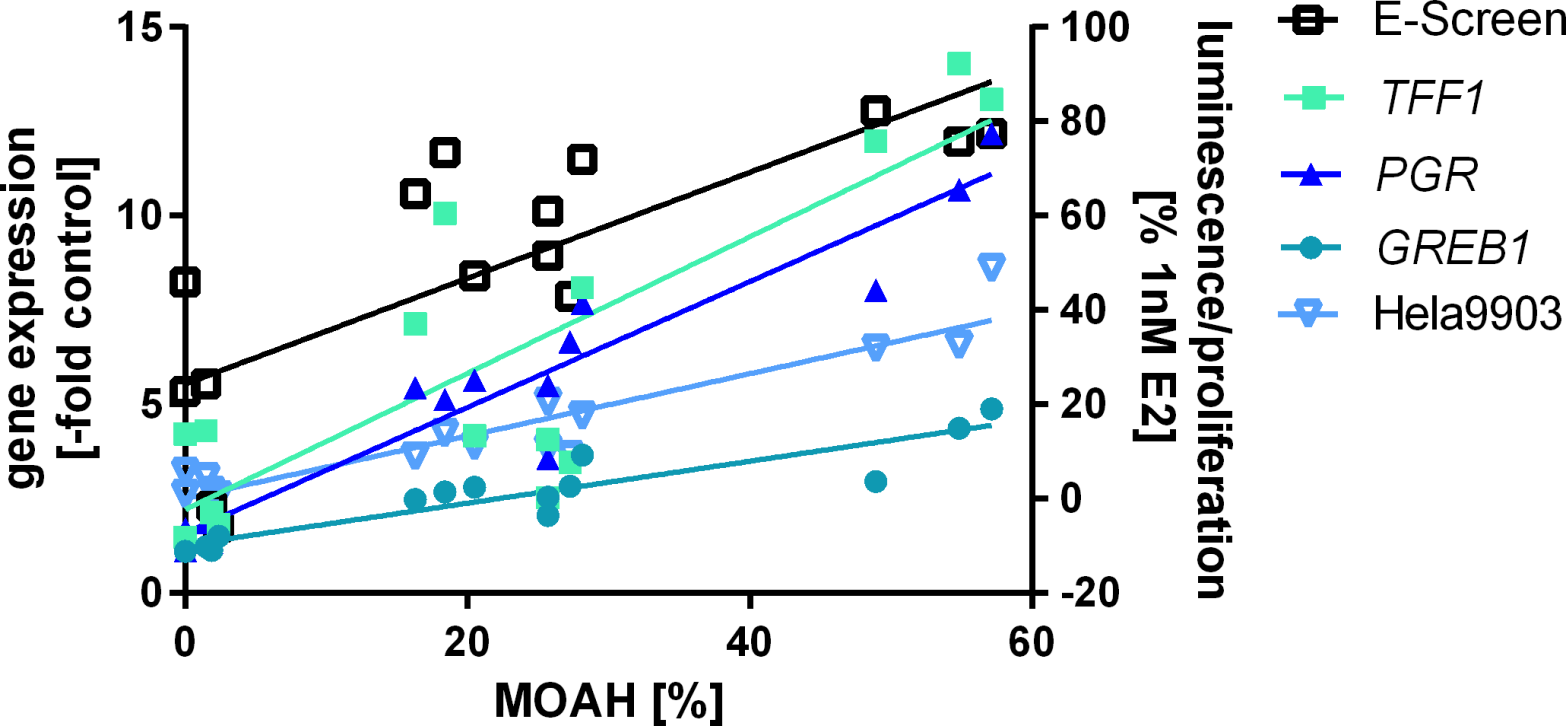
Correlation of estrogenic effects with MOAH percentage. Results from the estrogen assays correlated well with the percentage of MOAHs being present in MOs. For the E-screen data from exposure to 0.1 μl/ml MO was used.

To further characterize the fractions responsible for the observed xenoestrogenic activity the MOs were separated using HPLC (Table 1). The data confirmed high contents of MOAHs for most of the oils, with percentages as high as 57%. The MOAH fractions were then analyzed further via two-dimensional GC (data not shown, see S1 Fig for exemplary online-LC-GC-FID and GCxGC-ToF-MS chromatograms). The data highlighted differences in the composition of some of the MOAH fractions. These include, for example, exceptionally high-molecular weight compounds present in MO 4 as indicated by the later retention time in the melting point dimension of the two-dimensional GC. In addition high percentages of substituted PAHs were detected in MOs 9 and 13 (S1 Fig). Other components detected were alkylated benzenes, alkylated and partly hydrogenated naphthalenes and traces of unsubstituted or low alkylated three ring systems. However, although the online-LC-GC-FID and GCxGC-ToF-MS chromatograms of the respective MOAH fractions show a certain degree of similarity the spectral finger prints are quite different. Altogether, comparison of the analytical results with the xenoestrogenic activity showed a clear correlation of the latter with the percentage of MOAHs being present in the MO (Fig 2). This comprised gene expression levels (r^2^ = 0.91, 0.68 and 0.85 for *PGR*, *TFF1* and *GREB*, respectively) as well as data from the E-screen at 0.1 μl/ml and the luciferase assay (r^2^ = 0.61 and 0.87, respectively).

**Table 1:** Percentages of MOAHs and carbon range of MOSHs and MOAHs from the various MOs as determined by online-LC-GC-FID analysis. n.d. = not detectable.

MO	MOAHs [%]	MOSH range	MOAH range
1	27.3	C14-C24	C14-C24
2	25.7	C13-C28	C14-C29
3	n.d.	C10-C18	-
4	25.7	C13-C34	C13-C34
5	n.d.	<C16	-
6	2.4	C13-C22	C14-C21
7	1.9	C13-C20	C14-C21
8	1.5	C12-C22	C13-C18
9	20.5	C13-C21	C13-C21
10	16.3	C13-C20	C13-C26
11	18.4	C13-C21	C12-C22
12	28.1	C13-C23	C13-C22
13	57.1	C13-C23	C13-C23
14	54.8	C13-C22	C12-C21
15	48.9	C13-C21	C12-C20

### Estrogenic potential of MOSHs and MOAHs

**Fig 3:**
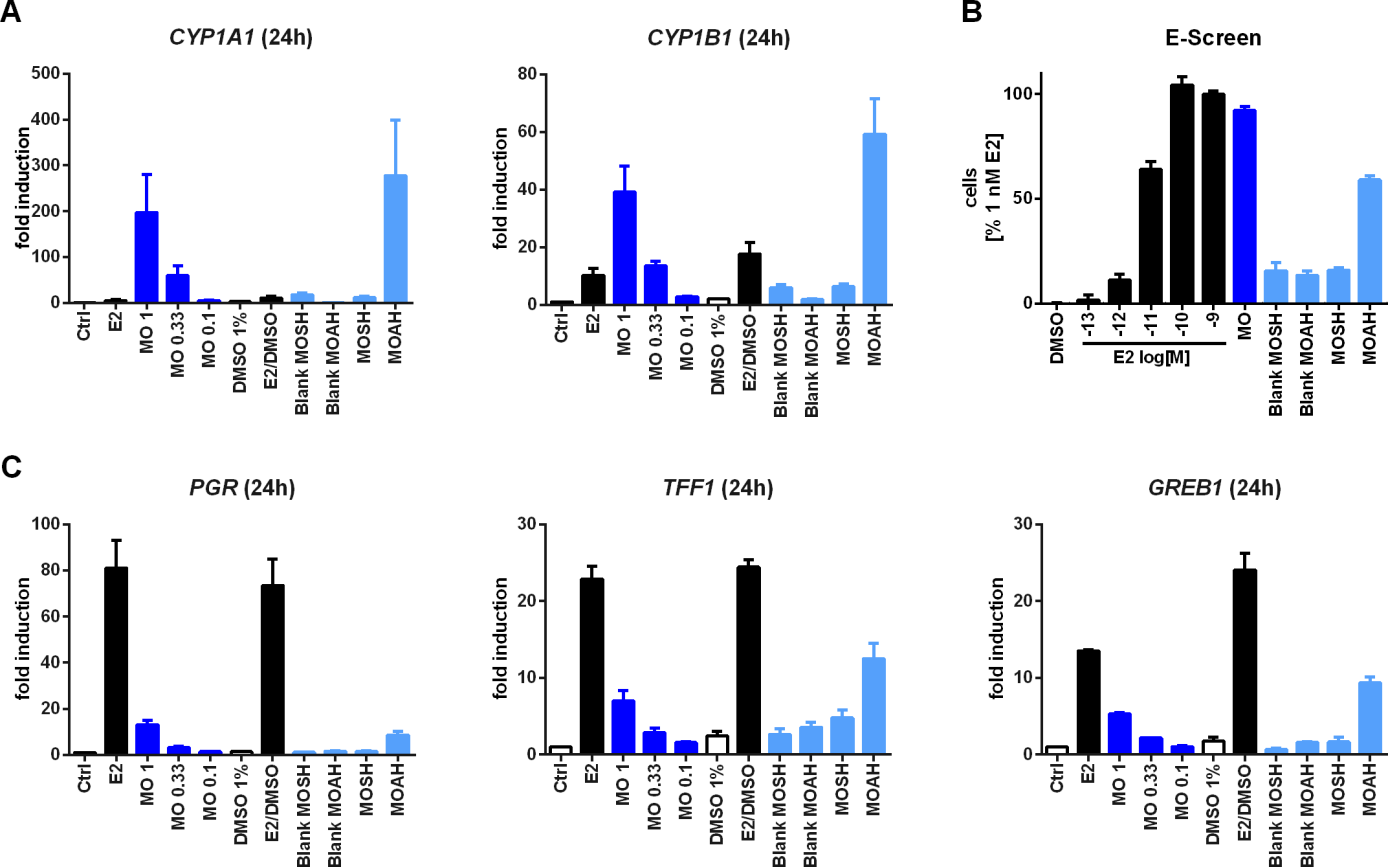
Analytical separation and subsequent analysis of MOSH and MOAH fractions from MO 1. Transcriptional analysis of *CYP1A1* and *1B1* was used as a biological control to establish separation of MOSHs and MOAHs (**A**), while the E-screen (**B**) and estrogenic activity-dependent gene transcription (**C**) are readouts for xenoestrogenic potential. If not stated otherwise concentrations of E2 [M] and MO [μl/ml] were used as indicated or added as 10 nM E2. For the E-screen MOSH and MOAH fractions were added in final concentrations of 14.6 and 5.4 μg/ml, respectively, while the concentrations for the gene expression analysis were 146 μg/ml and 54 μg/ml, respectively. When fractions were diluted 1:100 for the transcriptional assays, additional positive and negative controls were applied to account for the final DMSO concentration (DMSO 1% and E2/DMSO). Relative gene expression was normalized to *RPLP0* as reference gene and to the solvent control. Graphs represent mean ± SEM from 3 independent experiments. Abbreviations: *PGR*, progesterone receptor (gene); *TFF1*, trefoil factor (gene); *GREB1*, estrogen-dependent growth regulator in breast cancer 1 (gene); *CYP1A1/1B1* (gene), cytochrome P450-dependent monooxygenase, family 1, subfamily A/B, polypeptide 1.

The assumption that the xenostrogenicity of MOs is mediated by compounds of the MOAH fraction was tested further by comparing the endocrine activities of MOSHs and MOAHs from an exemplary oil, namely MO 1. The respective fractions were isolated by preparative HPLC using consecutive sample injections and a hexane/dichloromethane gradient. Preparations for negative controls consisted of mock hexane injections which were separated using the same program and are henceforth referred to as ‘blank MOSH’ and ‘blank MOAH’, respectively. Prior to subsequent experiments transcription of the AHR responsive genes *CYP1A1* and *1B1* was used as an additional biological indicator for assessing the preparations. Cells were exposed to 146 μg/ml MOSHs and 54 μg/ml MOAHs (Fig 3A). Only the MOAH-fraction led to a visible induction of CYP1-transcripts. While not being exclusive control this indicates the carry over of aromatic substances to be low to negligible, given the usually high sensitivity of AHR-regulon for aromatic substances. Likewise expression of *CYP1A2*, another AHR target gene, was only elevated following exposure to MOAH (S3 Fig). However, the respective expression levels were lower than for *CYP1A1* and *1B1*.

The estrogenic activity was tested using the E-screen (Fig 3B). The data show that the proliferative effect can be attributed to the MOAH fraction, with 5.4 μg/ml of MOAHs corresponding to about double-digit picomolar concentrations of E2. In contrast, exposure to MOSHs failed to significantly enhance cell proliferation at a concentration of 14.6 μg/ml when compared to the MOSH blank controls. Concomitantly the canonical estrogenic transcripts *PGR*, *TFF1* and *GREB* was also significantly induced by the MOAH fraction only (Fig 3C). The effect was less pronounced for the non-exclusively ERE-regulated *HSPB8* or *CTSD* (S3 Fig).

### Comparison of xenoestrogenic MOAH activity and evaluation vs. its genotoxic potential

**Fig 4:**
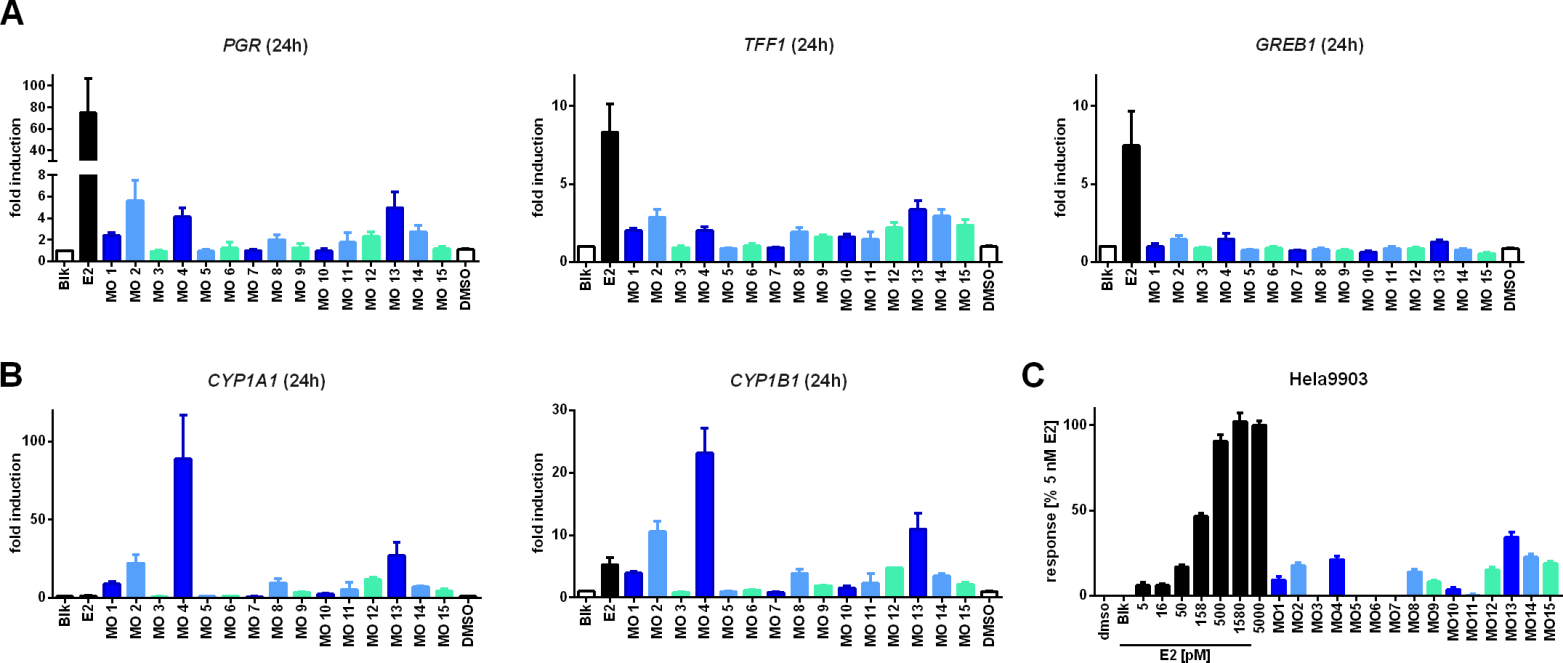
Analysis of the estrogenic potential of MOAHs. Activation of ER- (**A**) and AHR-dependent (**B**) gene transcription was measured following 24-h exposure to 1 μl/ml MOAHs or 10 nM E2, while luminescent reporter cells were subjected to 5 μl/ml MOAHs for 24 h (**C**). Data represent the mean ± SEM from at least three independent experiments. Again gene expression levels were normalized to the reference gene *RPLP0* and the solvent control, while luminescence values were corrected for the background activity of the solvent control and normalized using 5 nM E2 as maximal response. Blk (blank) represents the flow through of a mock sample which was treated similarly to the MOAH fractions. Abbreviations: *PGR*, progesterone receptor (gene); *TFF1*, trefoil factor (gene); *GREB1*, estrogen-dependent growth regulator in breast cancer 1 (gene); *CYP1A1/1B1* (gene), cytochrome P450-dependent monooxygenase, family 1, subfamily A/B, polypeptide 1.

Following the identification of MOAHs as a source for endocrine activity in MO 1, MOAH fractions of all oils were screened with regard to their potential to induce estrogenic activity-dependent gene transcription (Fig 4A) or to activate ERα (Fig 4C). It should be noted that in contrast to MO 1 these assays were performed using a ten-fold higher dilution since some MOs began showing signs of cytotoxicity or precipitated when used at higher concentrations (data not shown). Depending on the MOAH content this corresponded to up to 11.2 μg MOAH/ml, except for MO 3 and MO 5 which are MOAH-free and hence were tested using mock preparations instead. At the concentrations used none of the samples induced *GREB1*, *HSPB8* or *CTSD* (Fig 4 and S4 Fig). However, transcription of *PGR* and *TFF1* was found to be upregulated for several of the samples, particularly MOs 2, 4 and 13. The Hela-9903 transactivation assay confirmed these results with MO 13 being as potent as 50–100 pM E2, when MOAH fractions were applied in a 1:200 dilution, corresponding to a MOAH concentration up to 22.4 μg/ml (Fig 4C).

**Fig 5:**
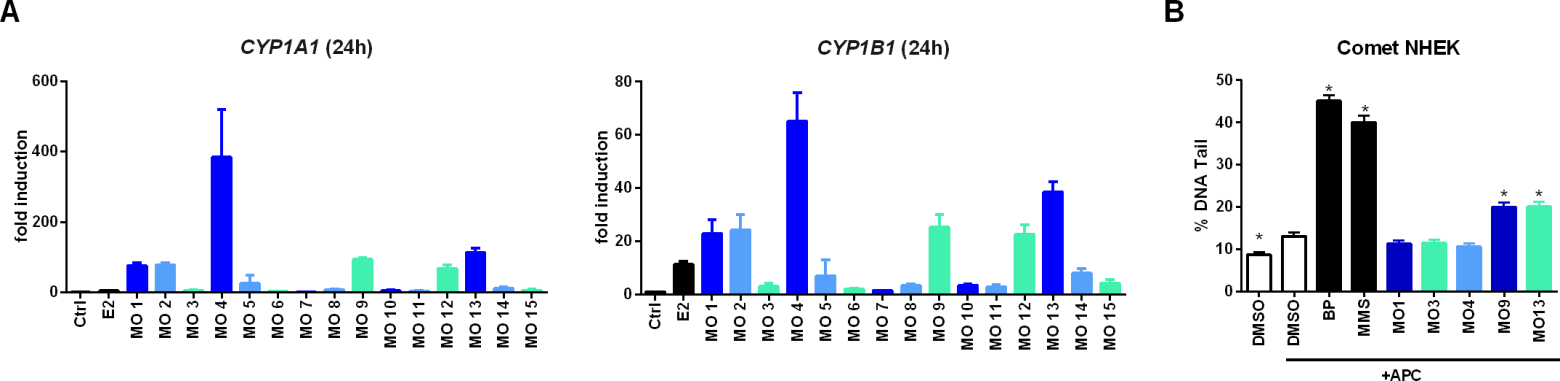
CYP induction and DNA damage caused by various MOs. Activation of AHR-dependent gene transcription in MCF-7 cells was measured following 24-h exposure to 1 μl/ml dispersed MO or 10 nM E2 (**A**). Data represent the mean ± SEM from at least three independent experiments. Gene expression levels were normalized to the reference gene *RPLP0* and the solvent control. For the comet assay (**B**), NHEKs were exposed to 1 μl/ml of dispersed MO, 3 μM benzo[*a*]pyrene (BP) or 20 μM methyl methanesulfonate (MMS) in presence of aphidicolin (APC). The resulting DNA damage was analyzed using an alkaline comet assay and quantified based on % DNA detected in the comet tails. Data represent the mean ± SEM of three independent experiments with cells from two individual donors resulting in 300 analyzed cells (* statistically significant (P<0.05) with respect to the APC/DMSO control as assessed by the Dunnett’s test). Abbreviations: *CYP1A1/1B1* (gene), cytochrome P450-dependent monooxygenase, family 1, subfamily A/B, polypeptide 1.

Likewise the MOAHs from MO 2, 4 and 13 acted as strong inducers for the *CYP1A1*, *1B1* and *1A2* (Fig 4B and S4 Fig). The corresponding fractions thus not only act as xenoestrogens but are likely to also influence phase I metabolism through AHR signaling. In case of other polycyclic aromatic compounds such as benzo[*a*]pyrene (BP) this can potentially lead to the formation of genotoxic metabolites [23, 24]. Given that genotoxicity is a major concern with regard to MOs used in printing inks all oils were screened for their potential to induce *CYP1A1 1B1* and 1A2 (Fig 5A, S2 Fig), followed by the comet assay for some selected oils. However, in comparison to the positive controls BP (DNA damaging upon CYP-dependent activation) and methyl methanesulfonate (MMS; directly alkylating), from the major CYP inducers only MO 9 and 13 showed some weak genotoxic potential (Fig 5B).

## Discussion

Given their widespread use and consumer exposure the toxicological properties of MOs in printing inks have been a matter of scientific and regulatory debate [8]. Yet, the focus has so far been systemic toxicity or genotoxicity rather than potential endocrine effects. This is the first time that MOs were systematically assessed as potential endocrine disruptors. Indeed the majority of oils were found to exert some estrogenic effects *in vitro*, be it on the transcriptional level, by activation of the ERα or by stimulating estrogen-dependent cell proliferation of MCF-7 cells. Thereby, the estrogenic activity correlates well with the percentage of MOAHs in the respective MOs, a finding that was confirmed further by separate analysis of the effects of the individual MOSH and MOAH fractions, respectively. However, compared to E2 MO-featured hormonal activity was subject to notable variation, particularly between the different assays. Although all assays used are based on human cell lines each assay is based on a different principle and is thus prone to inherent limitations. These are for example an increased risk for cytotoxicity as a consequence of prolonged exposure during the E-screen, the possibility of direct substance interactions with firefly luciferase in the reporter gene assay [15, 25], or unspecific transcriptional effects. While this necessarily limits the predictivity of each assay and explains the observed variability, it is unlikely that one and the same compound or compound mixture will produce false-positive readouts in several assays. Out of the 15 MOs tested 10 triggered estrogenic responses in all assays used, that is MOs 1, 2, 4, 8, 9, 10, 12, 13, 14 and 15. It should be noted that all of these MOs contained MOAHs at percentages of 16% or higher, whereas samples with lower amounts of MOAHs tended to be less clear in their response. Even though these results clearly indicate a potential hazard it is difficult to assess any estrogenic potency. In the E-screen several MOs produced effects equivalent to E2 at high picomolar concentrations. Yet, as a cellular assay this screen integrates various processes in the cell [26] and thus can be affected by cytotoxicity or physiological bias unrelated to estrogen signaling. Similarly the RNA levels of well-established estrogen responsive genes such as *TFF1* [27] can be subject to cross-regulation by phorbol esters and growth factors, the latter acting synergistically with E2 [28, 29]. Nevertheless, it should be noted that all MOs tested have a low solubility and were added to the assays at dilutions ranging from 1:1000 to 1:10,000. Moreover, the respective MOAH fractions are complex mixtures comprising several hundred different compounds. With only a fraction of these being likely to act as xenoestrogens the latter must be sufficiently potent in order to trigger a measurable signal.

Contrary to the situation seen with the genes controlled by the ER expression of the AHR target genes *CYP1A1* and *CYP1B1* did not generally correlate with MOAH contents (data not shown). This is exemplified by the fact that PAH-containing oils such as MO 4 induced a strong gene response, while other MOAH-rich oils like MO 14 and MO 15 failed to do so. The observed AHR activity of some oils prompted us to investigate genotoxic effects by applying a modified comet assay. As it is known for higher molecular weight PAHs such as BP, AHR activation induces CYP expression and thus conversion of PAHs to DNA reactive metabolites (*e*.*g*., diol epoxides). We indeed observed some genotoxic effects for some oils, however MO 4, which showed the highest AHR activation resp. CYP1 induction, did not induce DNA strand breaks or adducts leading to nucleotide excision repair detectable in the comet assay (Fig 5). In contrast MO 9 and MO 13, which induced a much weaker CYP expression compared to MO 4, significantly increased the percent tail DNA in the modified comet assay. MO 13 was also the oil with the highest MOAH content consisting mainly of alkylated naphthalenes and traces of alkylated three ring molecules. It has been shown, that naphthalene does not activate AHR in Hepa-1 cells and that AHR knockout mice are not protected from naphthalene-mediated toxicity [30]. Results concerning genotoxicity of naphthalene are controversial when *in vitro* and *in vivo* assays are compared [31]. Moreover, genetic toxicity of alkylated naphthalenes is scarcely investigated. However, for some higher molecular PAHs, the alkylated molecules showed an increased carcinogenic potency when compared to their non-alkylated counterparts [32, 33].

An activated AHR is known to potentially interfere with estrogen signaling. Known mechanisms comprise recruitment of shared transcriptional cofactors, upregulation of estrogen metabolizing enzymes or direct protein-protein interaction with ERs [34]. The latter not only results in the recruitment of ER to AHR target promotors and *vice versa* but also to proteasomal degradation of the ER, which in turn will inhibit estrogen signaling [35–38]. Thus estrogenic readouts can be dampened in mixtures with high AHR activity.

Most known (xeno)estrogens contain phenolic residues (*i*.*e*., bisphenol A, nonylphenols, diethylstilbestrol, parabens and benzophenones as well as the endogenous E2), which in fact seems to be a prerequisite for ER binding [39]. Hence one might speculate the MOAH fraction to contain phenolic xenoestrogens as such or some metabolic precursors thereof. Indeed it has been shown that some PAHs (*i*.*e*., BP or 3-methylcholanthrene) require hydroxylation prior to ER activation [40, 41], while others bind to the ERα directly [42]. Due to the high number of individual compounds present in MOs currently one can only but speculate about which classes of compounds are responsible for the estrogenic activities. The most active oil, MO 13, mainly contains alkylated benzenes and alkylated naphthalenes, but traces of three ring PAHs were also detected.

The MOAH-induced effects of MO 1 occurred at 54 mg/l and 5.4 mg/l (cf. Fig 3A and 3B). This corresponds to concentrations found in foodstuffs with ranges from more than 1 to 60 mg of MOAH/kg foodstuff, respectively [3]. Also, migration studies with packages made from recycled paperboard have shown that storage of several months will typically lead to 2.8–9.4 mg MOAH/kg dry food [4, 43]. Overall there are few reports regarding the estrogenic effects of MOAH-containing MOs. Vabrie *et al*. investigated the effects of crude and refined oils in MCF7 cells, reporting up-regulation of *TFF1* and increased cell proliferation at 25 mg/l [44]. Likewise experiments in zebrafish suggest that crude oils might affect the endocrine system by disrupting steroidogenesis [45]. However, the activation of hormone receptors has not been studied in this context nor has the role of MOs in previous studies on AHR activation and estrogenic potential in paper extracts [46, 47].

Altogether it appears that MOs used in printing inks contain potential endocrine disruptors. In absence of more detailed data on MO-composition, -kinetics and metabolism this has to be seen as a potential hazard ond one should assess carefully if and to what extent they might contribute to the total estrogenic burden in humans. Moreover, any comprehensive risk assessment of these oils will have to address their effects as mixtures and, ideally, establish the quantitative dose effect relationship of the single substances contained therein.

## Supporting Information

S1 Fig GCxGC-ToF-MS und online-LC-GC-FID chromatograms of 3 selected MOs. Isolated MOAH fractions were subjected to GCxGC-ToF-MS analysis (MO 4: A, MO 9: B, MO 13: C). The x-axis shows separation according to the boiling point, the y-axis separation due to polarity. The relative intensity is represented by the color scale. The regions for different classes of compounds (alkylated benzenes, naphthalenes and 3-ring systems) are marked in the chromatograms by a pink, green and orange grid, respectively. The chromatographic finger prints of naphthalene fractions and corresponding mass spectra clearly indicate partial hydrogenation of constituents in all MOs shown (MO 4, 9 and 13). The corresponding online-LC-GC-FID chromatograms are depicted in part D (MO 4), E (MO 9), and F (MO 13), respectively. They reveal characteristic fingerprints for each oil and highlight the higher molecular components of MO 4 in contrast to MO 9 and MO13.
(TIF)

S2 Fig Effects of MOs on ER or AHR responsive transcripts. Hormone starved MCF7 cells were treated with 1 g/l MO or 10 nM E2 for 24 h. Gene expression was quantified by Real-Time PCR. Data in all assays represent the mean ± SEM from at least three independent experiments. Gene expression was normalized using *RPLP0* and the solvent control as references. Abbreviations: *HSPB8*, heat shock protein family B (small) member 8 (gene), *CTSD*, cathepsin D (gene), *CYP1A2*, cytochrome P450 family 1 subfamily A member 2 (gene).
(TIF)

S3 Fig Effects of MOSH and MOAH fractions from MO 1 on ER and AHR responsive transcripts. MO1 was diluted as indicated [μl/ml]. Fractions were diluted 1:100 and additional positive and negative controls were applied to account for the final DMSO concentration (DMSO 1% and E2/DMSO). Relative gene expression was normalized to *RPLP0* as reference gene and to the solvent control. Graphs represent mean ± SEM from 3 independent experiments. Abbreviations: *HSPB8*, heat shock protein family B (small) member 8 (gene), *CTSD*, cathepsin D (gene), *CYP1A2*, cytochrome P450 family 1 subfamily A member 2 (gene).
(TIF)

S4 Fig Effects of MOAH fractions from different MOs on ER and AHR responsive transcripts. Activation of ER- and AHR-dependent gene transcription was measured following 24-h exposure to 1 μl/ml MOAHs or 10 nM E2. Data represent the mean ± SEM from at least three independent experiments. Again gene expression levels were normalized to the reference gene *RPLP0* and the solvent control. Abbreviations: *HSPB8*, heat shock protein family B (small) member 8 (gene), *CTSD*, cathepsin D (gene), *CYP1A2*, cytochrome P450 family 1 subfamily A member 2 (gene).
(TIF)
