# Supplementary figures and images for: Estrogenic Activity of Mineral Oil Aromatic Hydrocarbons Used in Printing Inks
Source: PLoS One. 2016 Jan 15;11(1):e0147239. doi: 10.1371/journal.pone.0147239 (PMC4714758; doi:10.1371/journal.pone.0147239)

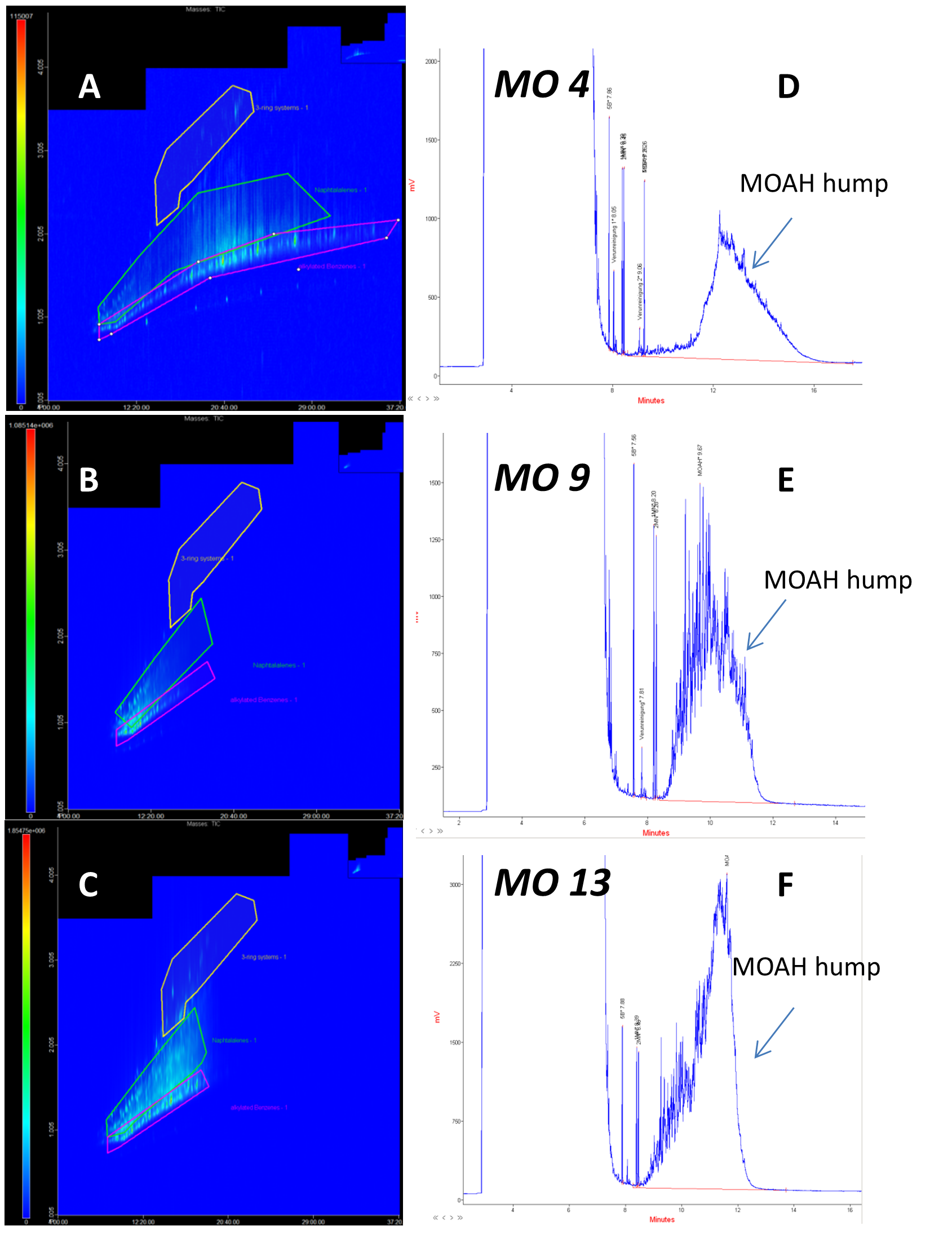

Supplement: S1 Fig — Isolated MOAH fractions were subjected to GCxGC-ToF-MS analysis (MO 4: A, MO 9: B, MO 13: C). The x-axis shows separation according to the boiling point, the y-axis separation due to polarity. The relative intensity is represented by the color scale. The regions for different classes of compounds (alkylated benzenes, naphthalenes and 3-ring systems) are marked in the chromatograms by a pink, green and orange grid, respectively. The chromatographic finger prints of naphthalene fractions and corresponding mass spectra clearly indicate partial hydrogenation of constituents in all MOs shown (MO 4, 9 and 13). The corresponding online-LC-GC-FID chromatograms are depicted in part D (MO 4), E (MO 9), and F (MO 13), respectively. They reveal characteristic fingerprints for each oil and highlight the higher molecular components of MO 4 in contrast to MO 9 and MO13. (TIF) [file pone.0147239.s001.tif]
